# Anticoagulant outcomes in managing tumor thrombus: a systematic review

**DOI:** 10.3389/fonc.2026.1773327

**Published:** 2026-02-23

**Authors:** Lama Alfehaid, Samaher Alatmi, Bishier Alfadhel, Razan Alqahtani, Shooa bin Nafisah, Yara Alotaibi, Mohammed AlSheef, Nada Alsuhebany

**Affiliations:** 1 Department of Pharmacy Practice, College of Pharmacy, King Saud bin Abdulaziz University for Health Sciences, Riyadh, Saudi Arabia; 2 Pharmaceutical Care Department, King Abdulaziz Medical City, Riyadh, Saudi Arabia; 3 King Abdullah International Medical Research Center, Riyadh, Saudi Arabia; 4 Department of Pharmacy Practice, College of Pharmacy, King Saud University, Riyadh, Saudi Arabia; 5 Pharmaceutical Care Department, King Saud University Medical City, Riyadh, Saudi Arabia; 6 King Fahad Medical City, Riyadh, Saudi Arabia; 7 College of Medicine, Alfaisal University, Riyadh, Saudi Arabia

**Keywords:** anticoagulation, bleeding risk, cancer-associated thrombosis, cardio-oncology, hepatocellular carcinoma, renal cell carcinoma, tumor thrombus

## Abstract

**Background:**

Tumor thrombus (TT), defined as intravascular extension of malignant tissue, is a distinct manifestation of cancer-associated thrombosis driven by malignant vascular invasion rather than fibrin-rich clot. Despite limited evidence, anticoagulant therapy is frequently prescribed empirically. This systematic review synthesized available data on the effectiveness and safety of anticoagulation in adults with solid tumor–associated TT.

**Methods:**

A PRISMA (2020) compliant systematic review (PROSPERO-registered) was conducted using PubMed/MEDLINE, Embase, and CENTRAL through September 2025. Eligible studies included adults with solid tumor–associated TT who received anticoagulation and reported thrombus response, thromboembolic recurrence, survival, or bleeding outcomes. Results were synthesized narratively in accordance with SWiM guidance. Risk of bias was assessed using the Newcastle–Ottawa Scale and the Joanna Briggs Institute tools, and the certainty of evidence was assessed using the GRADE approach.

**Results:**

Eight studies met the inclusion criteria, comprising three retrospective cohort studies and five case reports. Anticoagulants evaluated included low-molecular-weight heparin, vitamin K antagonists, direct oral anticoagulants, unfractionated heparin, and parenteral direct thrombin inhibitors. Across all studies, anticoagulation alone was not associated with radiographic regression of TT. Apparent thrombus resolution occurred exclusively following tumor-directed or mechanical interventions, such as surgical thrombectomy or percutaneous aspiration, and could not be attributed to anticoagulation alone. Evidence for a reduction in thromboembolic recurrence was limited, heterogeneous, and inconsistently adjudicated as bland thrombosis versus malignant embolic disease. No survival benefit attributable to anticoagulation was demonstrated. In contrast, anticoagulant therapy was associated with a clinically meaningful risk of major bleeding, particularly among patients with renal cell carcinoma–associated TT. Overall certainty of evidence ranged from very low to moderate.

**Conclusion:**

Current evidence does not demonstrate a consistent benefit of routine anticoagulation for isolated TT. Anticoagulation may be appropriate when conventional indications are present (e.g., pulmonary embolism, proximal deep-vein thrombosis, atrial fibrillation, catheter-associated thrombosis). Tumor-directed therapy remains the primary determinant of outcomes. Prospective studies are needed to define optimal management strategies.

**Systematic Review Registration:**

https://www.crd.york.ac.uk/PROSPERO/view/CRD420251111117, identifier CRD420251111117.

## Introduction

Venous thromboembolism (VTE), encompassing deep-vein thrombosis (DVT) and pulmonary embolism (PE), affects approximately 1.3 to 1.5 per 1,000 adults annually and remains a significant cause of preventable morbidity and mortality worldwide (1). Standard diagnostic modalities, including compression ultrasonography and computed tomography pulmonary angiography, allow timely detection, and anticoagulant therapies, such as heparins, vitamin K antagonists (VKA), and direct oral anticoagulants (DOACs), achieve high rates of efficacy in the general population (1–3).

Among patients with malignancy, however, the incidence, severity, and clinical consequences of thrombosis are substantially amplified (4). Cancer-associated thrombosis (CAT) is now recognized as the second leading cause of death in oncology patients, surpassed only by cancer progression itself (5). Tumor-related mechanisms—including tissue factor overexpression, release of procoagulant microparticles, tumor–platelet interactions, neutrophil extracellular trap formation, and cytokine-mediated endothelial dysfunction—create a profoundly prothrombotic milieu while simultaneously increasing bleeding risk and complicating anticoagulant management (6).

A distinct and less common manifestation within the spectrum of CAT is tumor thrombus (TT), defined by direct intraluminal extension of malignant tissue into adjacent venous structures, such as renal vein or inferior vena cava involvement in renal cell carcinoma (RCC), or portal and hepatic vein invasion in hepatocellular carcinoma (HCC) (5). For the purposes of this review, TT is defined as an intravascular extension of malignant tissue, including contiguous macrovascular invasion (e.g., renal vein or inferior vena cava extension in RCC, portal or hepatic vein invasion in HCC), intracardiac tumor extension, and intravascular tumor emboli with confirmed malignant histology (7, 8). Unlike bland VTE, TT consists predominantly of viable malignant tissue interwoven with fibrin and platelets and is therefore biologically distinct from fibrin-rich thrombus (7, 9). Although these entities differ in anatomical location and clinical presentation, they share a common mechanism of malignant intravascular invasion and represent a unique pathophysiologic process within the spectrum of CAT.

In clinical practice, anticoagulant therapy is frequently prescribed in patients with TT based on extrapolation from CAT management paradigms. However, whether anticoagulation provides meaningful clinical benefit in TT, such as thrombus regression, prevention of embolic events, or survival improvement, remains uncertain. The available evidence is sparse, heterogeneous, and confounded by advanced malignancy, vascular invasion, and competing bleeding risks (10). As a result, major society guidelines offer only limited and sometimes conflicting recommendations regarding anticoagulation in TT, reflecting variability in tumor biology, thrombus extent, and available data (11, 12). Although recent observational studies and narrative reviews have highlighted substantial practice variability and the increasing use of DOACs, the comparative effectiveness and safety of anticoagulation in this population have not been systematically evaluated (9, 10, 13). Importantly, patients with TT have been systematically excluded from major randomized trials of CAT, including studies evaluating low-molecular-weight heparins and direct oral anticoagulants. As a result, the applicability of CAT trial evidence to patients with malignant intravascular invasion remains uncertain, and current anticoagulation practice for tumor thrombus is largely extrapolated from CAT paradigms, with limited direct supporting data. This evidence gap creates substantial clinical uncertainty regarding the effectiveness and safety of anticoagulation in this distinct population (14–16).

To date, no comprehensive systematic review has synthesized the available evidence addressing anticoagulant use specifically in solid tumor–associated tumor thrombus. Addressing this gap is critical to clarify the potential benefits and harms of anticoagulation in TT, inform multidisciplinary clinical decision-making, and guide future research and guideline development. Therefore, this systematic review aims to critically appraise and synthesize available studies evaluating the impact of anticoagulant therapy on thrombus resolution, thromboembolic recurrence, survival, and bleeding outcomes in adults with solid tumor–associated tumor thrombus.

## Methods

### Review design and registration

This systematic review was conducted in accordance with the Preferred Reporting Items for Systematic Reviews and Meta-Analyses (PRISMA 2020) guidelines. The review protocol was prospectively registered in the International Prospective Register of Systematic Reviews (PROSPERO) under the title “Anticoagulant Outcomes in Managing Tumor Thrombus” (registration number: [CRD420251111117]). The methodological framework was adapted from the Cochrane Handbook for Systematic Reviews of Interventions.

### Eligibility criteria

Eligibility criteria were defined using the Population, Intervention, Comparison, and Outcome (PICO) framework (**Table 1**).

**Table 1:** Eligibility criteria were defined using the PICO framework.

Element	Criteria
Population	Adult patients (≥18 years) with a confirmed diagnosis of *tumor thrombus (TT)* secondary to solid malignancies (e.g., renal cell carcinoma [RCC], hepatocellular carcinoma [HCC], sarcoma, or melanoma).
Intervention	Any form of anticoagulant therapy administered for TT, including low molecular weight heparin (LMWH), VKAs, and DOACs.
Comparator	No anticoagulation, placebo, or standard oncologic/surgical care alone.
Outcomes	Primary outcomes included (1) thrombus reduction or resolution, (2) recurrence of thromboembolic events, (3) overall survival or mortality, and (4) incidence of major or clinically relevant bleeding. Secondary outcomes included patterns of anticoagulant use and concomitant oncologic or surgical interventions.
Study Types	Randomized controlled trials (RCTs), prospective and retrospective cohort studies, and case-control studies. Case reports and series were included for rare presentations where higher-level evidence was unavailable.
Exclusion Criteria	Pediatric populations, studies without a confirmed TT diagnosis, studies evaluating anticoagulation for non-tumor thrombi (e.g., bland thrombus), or those not reporting any prespecified outcomes.

### Information sources and search strategy

Comprehensive searches were conducted in the following electronic databases from inception through September 2025:

- PubMed/MEDLINE
- Embase
- Cochrane Central Register of Controlled Trials (CENTRAL)

The search strategy combined controlled vocabulary (MeSH) and free-text terms related to “tumor thrombus,” “cancer-associated thrombosis,” and “anticoagulants. “Search strategies available in **Supplementary Material**. Hand-searching reference lists was performed for key journals (JACC: CardioOncology, Thrombosis and Hemostasis, Clinical Oncology), and citation chaining was applied using Google Scholar to capture gray or recent literature.

### Study selection

All retrieved citations were imported into Mendeley^®^, and duplicates were removed. Three reviewers independently screened titles and abstracts for relevance, followed by a full-text assessment of potentially eligible studies. Discrepancies were resolved through discussion or consultation with a third reviewer.

### Data extraction and management

Data were independently extracted by three reviewers using a standardized Excel extraction sheet developed *a priori* (**Table 2**). Extracted data included:

**Table 2:** Summary of included studies (n=8).

Author (Year)	Tumor type	Tumor thrombus phenotype & location	Study design & Sample size	Anticoagulant regimen & dose	Imaging modality/TT assessment	Concurrent tumor-directed interventions	Efficacy outcomes (Regression, VTE, Survival)	Bleeding outcomes & definitions	Certainty of evidence (GRADE)
Balcar et al. (2024) (19).	HCC	Macrovascular invasion: portal and/or hepatic vein TT	Retrospective cohort (n=124)	DOACs (n=17), LMWH (n=2), VKA (n=5); dose per treating team (therapeutic intent)	CT and MRI every 3 months, and the results were read by 2 blinded radiologists	Systemic oncologic therapy; portal-HTN care (e.g., variceal management)	No TT regression; no survival benefit; numerical reduction in non-malignant VTE (NS; events not adjudicated as bland vs malignant)	Variceal bleeding lower in AC group; “major bleeding” study-reported (definition not extractable here)	⬤◯◯◯ (low)
Kaptein et al. (2022) (13).	RCC	Contiguous venous extension: renal vein → IVC (T-stage–aligned groups)	Retrospective cohort	LMWH or VKA (therapeutic)	TT defined by CT features (enhancement after contrast, vein expansion, growth despite AC) and/or histopathology; VTE confirmed by CT/MR/US; endpoints adjudicated by experts	Surgical thrombectomy common	No radiographic TT regression attributable to AC; higher VTE risk in TT; AC did not prevent VTE; higher mortality (confounded by stage/burden)	Major bleeding (MB) reported; study-defined and adjudicated	⬤⬤◯◯ (moderate)
Spelde et al. (2017) (20).	RCC	IVC TT extending above the diaphragm (cavo-atrial)	Case report (1 pt)	UFH peri-op → warfarin post-op (therapeutic)	Pre-op staging emphasizes contrast-enhanced CT and MRI as gold standards; intra-op surveillance includes TEE (case-based peri-op management)	Renal artery embolization; radical nephrectomy; caval thrombectomy (CPB/DHCA)	TT cleared after surgery; peri-op tumor PE; recovery (no inference for AC effect)	Not reported	⬤◯◯◯ (very low)
Führer et al. (2023) (21).	Urothelial carcinoma (reported as pulmonary tumor embolism syndrome)	Pulmonary tumor embolism with TT “in transit”	Case report (1 pt)	UFH → DOAC (therapeutic; adjunct)	Not specified in the extracted text available here	AngioVac aspiration; chemotherapy (gemcitabine/carboplatin)	Pathologic clearance after aspiration; AC adjunctive; died of progressive malignancy	Not reported	⬤◯◯◯ (very low)
Barron & Moon (2023) (22).	Malignant melanoma	Intracardiac TT (“ball-valve obstruction”)	Case report (1 pt; abstract)	Short-course heparin (~9 days; therapeutic)	Not specified in the abstract text available here	Surgical excision; cryoablation	Obstruction resolved after surgery; no AC-only response	Not reported	⬤◯◯◯ (very low)
Khajeh-Mehrizi et al. (2025) (23).	Renal Ewing sarcoma	IVC TT extending to right atrium	Case report (1 pt)	Rivaroxaban (initial; therapeutic intent)	Imaging performed with contrast-enhanced CT showing renal mass with IVC extension; TT extension described to RA	Cardiac surgery + nephrectomy; chemotherapy (VDC/IE)	TT cleared after surgery; short follow-up alive	Not reported	⬤◯◯◯ (very low)
Espiritu et al. (2002) (24).	IVC leiomyosarcoma (APS)	Malignant IVC obstruction (“tumor thrombosis”)	Case report (1 pt)	Warfarin → heparin → lepirudin/danaparoid ± IVIG/plasmapheresis	TT described on MRI/MRA of abdomen; venography used during evaluation/progression	Medical multimodal therapy	Progressive disease; mortality (disease-driven)	Not standardized	⬤◯◯◯ (very low)
Marcoux et al. (2019) (25).	RCC & HCC (majority)	Portal vein, renal vein, and/or IVC TT	Retrospective observational cohort (n=153; AC n=41)	AC used (class/dose not specified)	Inclusion required TT documented by CT, MRI, or US	Variable oncologic/surgical care (not standardized)	6-mo VTE incidence; 6-mo mortality; survival NS	Major bleeding reported; definition not provided in abstract	⬤◯◯◯ Very Low Certainty

AC, anticoagulation; APS, antiphospholipid syndrome; CPB, cardiopulmonary bypass; CT, computed tomography; DHCA, deep hypothermic circulatory arrest; DOAC, direct oral anticoagulant; HCC, hepatocellular carcinoma; HTN, hypertension; IVC, inferior vena cava; LMWH, low–molecular-weight heparin; MB, major bleeding; MRI, magnetic resonance imaging; MRA, magnetic resonance angiography; NS, not statistically significant; PE, pulmonary embolism; peri-op, peri-operative; intra-op, intra-operative; post-op, post-operative; pt, patient; RA, right atrium; RCC, renal cell carcinoma; TEE, transesophageal echocardiography; TT, tumor thrombus; UFH, unfractionated heparin; US, ultrasound; VDC/IE, vincristine, doxorubicin, cyclophosphamide alternating with ifosfamide and etoposide; VKA, vitamin K antagonist; VTE, venous thromboembolism.

This table summarizes published clinical studies and case reports evaluating the role of anticoagulation in patients with solid tumors complicated by tumor thrombus (TT). Tumor type, thrombus phenotype and anatomic location, study design, anticoagulant regimen and dosing strategy, imaging modalities used for TT assessment, concurrent tumor-directed interventions, efficacy outcomes (tumor thrombus regression, VTE, and survival), and bleeding outcomes are reported. Imaging-based TT diagnosis was primarily performed using contrast-enhanced CT and/or MRI, with expert adjudication where available. Bleeding outcomes are reported according to study-defined definitions when specified.

- Bibliographic information (author, year)
- Study design and setting
- Patient demographics and primary tumor type
- Anticoagulant type, dose, and duration
- Concomitant therapies (surgery, chemotherapy, embolization)
- Outcomes: TT resolution, thromboembolic recurrence, survival, and bleeding events
- Follow-up duration and study limitations

Disagreements were resolved by consensus. Where critical data were missing, the study authors were contacted for clarification.

### Quality and risk of bias assessment

Risk of bias was assessed using validated tools according to the study design: observational studies: the Newcastle–Ottawa Scale (NOS) for cohort and case-control designs; case reports/series: the Joanna Briggs Institute (JBI) critical appraisal checklist. Selective non-reporting bias and potential publication bias were further evaluated using the ROB-ME (“Risk of Bias due to Missing Evidence”) tool.

Three reviewers independently assessed each study, and a fourth investigator adjudicated discrepancies. The overall certainty of evidence for each key outcome was graded using the Grading of Recommendations Assessment, Development and Evaluation (GRADE) approach, downgrading for risk of bias, inconsistency, indirectness, imprecision, or publication bias.

### Outcome definitions and reporting

Bleeding outcomes were reported according to definitions used in the original studies. When provided, major bleeding was defined using study-specific criteria, most commonly aligned with International Society on Thrombosis and Hemostasis (ISTH) definitions (17, 18) or institutional adjudication standards. When bleeding definitions were not specified by the original authors, this was explicitly noted. Clinically relevant bleeding was defined according to study-specific criteria when available; otherwise, events were reported descriptively as clinically significant bleeding without standardized adjudication.

VTE recurrence was defined according to the original study definitions and reporting frameworks. When studies did not distinguish between bland thrombosis and malignant embolic phenomena, recurrence events were reported as described by the original authors, and lack of adjudication was explicitly acknowledged.

TT resolution or regression was defined as radiographic reduction or disappearance of intravascular tumor extension as reported by the original study. When thrombus regression occurred in the context of concurrent tumor-directed or mechanical interventions, this was explicitly noted and interpreted as not attributable solely to anticoagulation.

### Data synthesis and analysis

Due to anticipated clinical and methodological heterogeneity among included studies (tumor types, anticoagulant regimens, and outcome definitions), quantitative meta-analysis was deemed infeasible. Therefore, a narrative synthesis was conducted following the Synthesis Without Meta-Analysis (SWiM) reporting guideline.

Studies were grouped by:

- Primary tumor type (e.g., RCC, HCC, sarcoma, melanoma)
- Anatomic extent of TT (e.g., renal vein, IVC, suprahepatic)
- Anticoagulant class (LMWH, VKA, DOAC)

Within each subgroup, direction of effect and consistency across outcomes were tabulated and visually summarized. Median effect sizes and ranges were reported where feasible.

Certainty of evidence for each outcome domain was synthesized and summarized in evidence tables (**Table 2**).

## Results

### Overview of included studies

**Figure 1:**
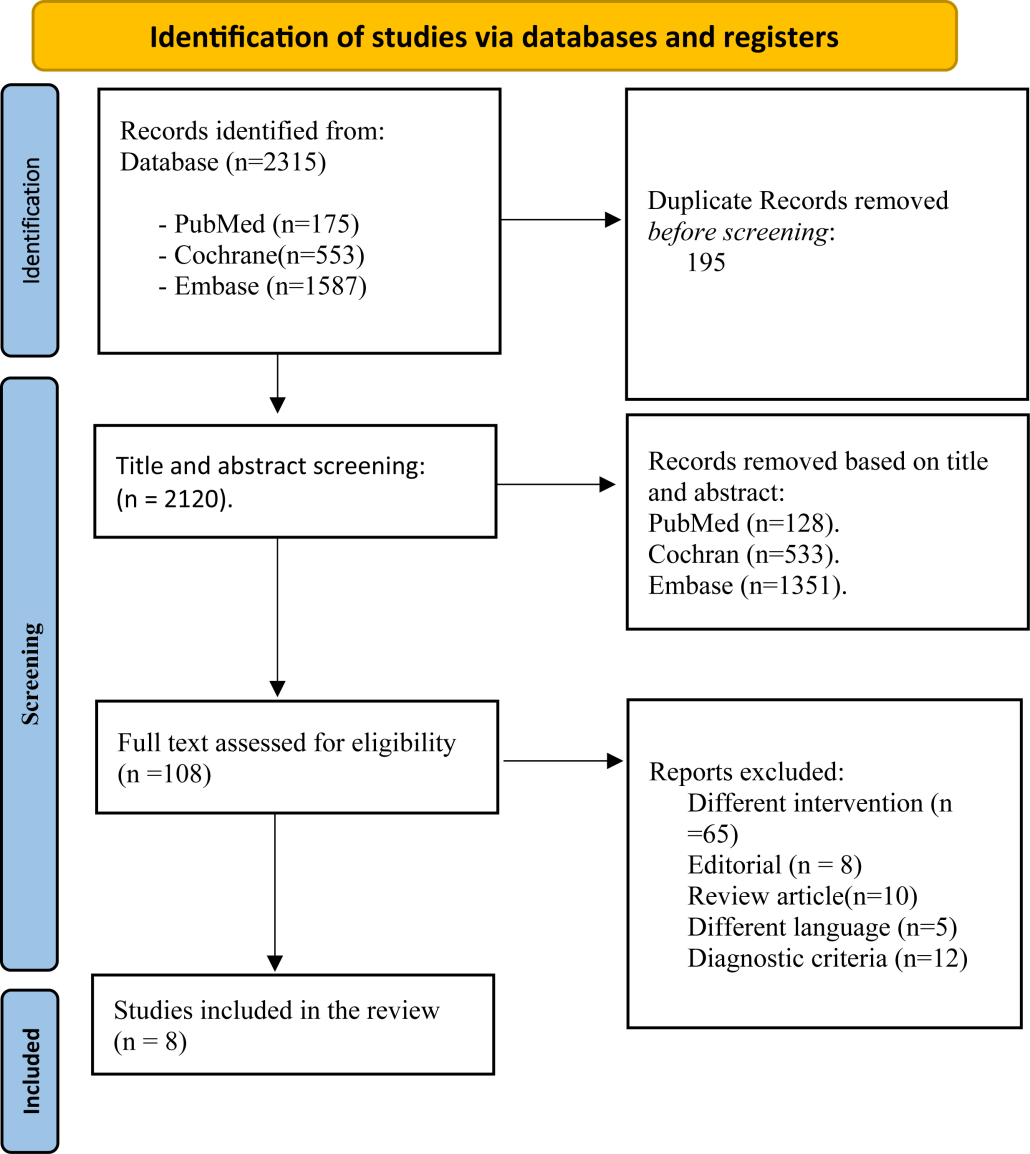
PRISMA flow diagram for the systematic review conducted, including (n=8) articles.

The database search identified 2,315 records (PubMed: n=175; Embase: n=1,587; Cochrane: n=553). After removing 195 duplicates, 2,120 unique citations underwent title and abstract screening. Of these, 2,012 were excluded for failing to meet the inclusion criteria. A total of 108 full-text articles were assessed, of which 100 were excluded primarily for evaluating non-anticoagulant interventions (n=65), being reviews outside predefined designs (n=10), lacking outcome data (n=12), being editorials (n=8), or written in non-English languages (n=5). Ultimately, eight studies met all criteria and were included in this systematic review. A PRISMA flow diagram summarizes the process (**Figure 1**).

### Characteristics of included studies

The eight included studies comprised three retrospective cohorts and five case reports. One study was available only as a conference abstract (Marcoux et al.), while the remaining studies were full-text publications. Collectively, the included studies reported patients with tumor thrombus arising from RCC, HCC, melanoma, urothelial carcinoma, Ewing sarcoma, and leiomyosarcoma. Sample sizes ranged from single-patient case reports to cohorts of up to approximately 700 patients with RCC. Anticoagulants evaluated included LMWH, VKAs, DOACs, UFH, and, in rare cases, parenteral direct thrombin inhibitors (e.g., lepirudin, danaparoid). Co-interventions frequently included radical nephrectomy with thrombectomy, AngioVac aspiration, embolization procedures, variceal management, and systemic anticancer therapies. **Table 2** summarizes the characteristics of the included studies.

### Patterns of anticoagulant use

Across studies, anticoagulation was rarely administered as monotherapy. Instead, it was typically used as an adjunct to multimodal management. LMWH was commonly used in RCC-related TT. DOACs were studied in three studies, primarily rivaroxaban or apixaban, in HCC and sarcoma. VKAs were often used in combination with UFH bridging. Despite this diversity, no study reported standardized dosing, duration, or criteria for initiating anticoagulation in TT, reflecting substantial practice variability.

### Effectiveness outcomes by tumor type and location

#### RCC with renal vein or inferior vena cava tumor thrombus

RCC–associated TT was the largest and most consistently reported subgroup and served as the primary source of cohort-level evidence. In the largest retrospective cohort study (Kaptein et al., 2022), patients with RCC and TT had substantially higher rates of venous thromboembolism (VTE) compared with RCC patients without TT. Although anticoagulation was frequently prescribed, it did not meaningfully reduce thromboembolic recurrence, and recurrent events were not adjudicated as bland thrombosis versus malignant embolic phenomena. Anticoagulation was also associated with increased mortality; however, this association was confounded by more advanced tumor stage and greater thrombus burden in anticoagulated patients. Radiographic thrombus regression attributable to anticoagulation was not reported. All documented thrombus clearance occurred following surgical thrombectomy. These findings suggest that in RCC, TT behavior is driven primarily by tumor biology and extent of vascular invasion rather than by anticoagulant exposure. Imaging modalities used to assess thrombus burden varied across studies (computed tomography, magnetic resonance imaging, and echocardiography), and the temporal relationship between imaging and intervention was inconsistently reported, limiting standardized interpretation of thrombus response.

#### HCC with portal or hepatic vein tumor thrombus

In HCC, TT reflects aggressive macrovascular invasion in the setting of cirrhosis and portal hypertension, representing a biologically and clinically distinct entity from RCC-associated TT. In the cohort study by Balcar et al. (2024), anticoagulation was not associated with thrombus regression or improved survival. A numerical reduction in non-malignant thrombotic events was observed, but did not reach statistical significance, and recurrence definitions were heterogeneous and not adjudicated as bland versus malignant embolic events.

Stabilization of thrombus was occasionally reported but occurred in the context of concurrent oncologic therapy and portal hypertension management and therefore cannot be causally attributed to anticoagulation. Assessment of thrombus burden relied on serial cross-sectional imaging, but imaging modality and timing relative to intervention varied, further limiting standardized interpretation of thrombus response. These findings highlight the dominant influence of tumor stage and liver disease severity on outcomes in this population.

#### Intracardiac tumor thrombus

Intracardiac TT was described exclusively in isolated case reports involving melanoma and RCC. These reports are presented descriptively to illustrate clinical presentation and management rather than to infer treatment effect. In all cases, anticoagulation was used only transiently and was followed by definitive mechanical removal via surgical excision or cryoablation. Thrombus resolution occurred exclusively following mechanical intervention, and no case demonstrated a response to anticoagulation alone. In these reports, thrombus burden was assessed using echocardiography or cardiac imaging at variable time points, and the temporal relationship between anticoagulation exposure and intervention was not standardized.

#### Pulmonary tumor embolism and tumor thrombus in transit

Pulmonary tumor embolism represents a distinct clinical entity characterized by malignant obstruction of the pulmonary vasculature. In the single reported case, thrombus resolution was achieved through AngioVac aspiration followed by chemotherapy. Anticoagulation was administered adjunctively but could not be isolated as the therapeutic driver of thrombus resolution. Imaging assessment relied on pulmonary angiography and computed tomography, with thrombus response occurring only after mechanical and oncologic intervention. As with intracardiac TT, this case is presented for descriptive purposes and does not permit inference regarding anticoagulant effectiveness.

#### Concomitant bland thrombosis

Several studies acknowledged the coexistence of bland thrombosis in patients with TT, particularly in RCC. However, subgroup outcomes were not systematically reported, and thromboembolic events were not consistently adjudicated as bland thrombosis versus malignant embolic phenomena. Imaging criteria used to distinguish bland from malignant thrombus were inconsistently reported, limiting the ability to draw conclusions regarding differential benefit from anticoagulation in patients with mixed malignant and non-malignant thrombotic components.

### Major and clinically relevant bleeding

Five studies reported bleeding events, although bleeding definitions varied and were not uniformly standardized across studies. Overall, anticoagulation was associated with a clinically meaningful bleeding risk. In the largest cohort study, Kaptein et al. (2022) observed a significantly higher risk of major bleeding among anticoagulated RCC patients, as defined by the original study criteria (hazard ratio 3.44), highlighting the vulnerability of this population. Marcoux et al. (2019) reported five major bleeding events among anticoagulated patients with RCC-and HCC-associated TT, although the bleeding definition was not explicitly specified.

In HCC, Balcar et al. (2024) reported no major gastrointestinal bleeding among anticoagulated patients and observed lower rates of variceal bleeding; however, this finding was likely attributable to aggressive prophylactic variceal management rather than a protective effect of anticoagulation itself.

Across case reports, no major bleeding events were described; however, follow-up was limited, and bleeding definitions were not standardized. These reports should therefore be interpreted cautiously.

### Other adverse events

Non-bleeding adverse events were infrequently reported. In the most complex case (Espiritu et al., 2002), a patient with antiphospholipid syndrome and leiomyosarcoma required multiple anticoagulant switches and adjunctive therapies. Reported complications were related to progressive malignancy and thrombus burden rather than to anticoagulant toxicity.

### Quality and certainty of evidence

Cohort studies scored 7/9 on the Newcastle–Ottawa Scale, indicating good methodological quality but limited adjustment for confounders. Case reports generally scored moderate to high quality on JBI criteria. Certainty of evidence (GRADE) ranged from very low to moderate, driven by small sample sizes, heterogeneity, and risk of bias. Across all outcomes, the overall certainty of evidence for anticoagulation in TT remained low, and conclusions should be interpreted cautiously.

## Discussion

This systematic review represents the first comprehensive synthesis of available evidence evaluating the effectiveness and safety of anticoagulant therapy in adults with solid tumor–associated TT. Several large surgical and oncologic series describing TT were identified but were excluded because they did not evaluate anticoagulation exposure or report anticoagulant-related outcomes. By systematically appraising eight studies encompassing multiple malignancies and anticoagulant classes, our findings provide a unified assessment of a clinical practice that remains widespread despite minimal empirical support.

Collectively, the included studies demonstrate that anticoagulation alone does not result in radiographic regression of TT, nor does it consistently protect against thromboembolic recurrence or improve survival outcomes. In contrast, a clinically meaningful risk of bleeding, particularly in patients with RCC, was repeatedly observed (14–21). Together, these findings suggest a limited therapeutic role for anticoagulation in modifying TT biology while highlighting a non-trivial safety burden.

A central and consistent observation of this review is the absence of TT regression attributable to anticoagulant therapy. All documented cases of complete thrombus resolution occurred exclusively following tumor-directed or mechanical interventions, including surgical thrombectomy, percutaneous aspiration (e.g., AngioVac), cryoablation, embolization, or systemic oncologic therapy (16–19). These observations reinforce the biological distinction between TT and bland venous thromboembolism: TT consists predominantly of viable malignant tissue rather than fibrin-rich clot, rendering it intrinsically resistant to anticoagulant-mediated dissolution (8, 10). Our synthesis therefore provides empirical confirmation, across tumor types and anticoagulant classes, that anticoagulation should not be expected to modify TT biology.

The evidence regarding thromboembolic recurrence was limited and heterogeneous. While TT was consistently associated with a higher baseline risk of VTE, most notably in RCC, anticoagulation did not reliably mitigate this risk. In the largest cohort study, anticoagulated patients with RCC-related TT continued to experience high rates of VTE despite treatment (15), suggesting that malignant embolic risk may be driven more by tumor burden and vascular invasion than by hypercoagulability alone. Data from HCC were similarly inconclusive, with numerical trends toward fewer non-malignant thrombotic events failing to reach statistical significance and remaining confounded by concurrent interventions (14, 21).

An important distinction in the management of patients with TT is between prophylactic-dose anticoagulation administered for routine cancer-associated VTE prevention and therapeutic-dose anticoagulation prescribed for established thrombosis or empirically for TT itself. These strategies serve different clinical objectives and should not be conflated. Most included studies evaluated therapeutic anticoagulation, and data on thromboprophylaxis in patients with TT were sparse and not systematically reported. As a result, current evidence does not permit conclusions regarding whether prophylactic anticoagulation prevents progression from localized tumor thrombus to systemic VTE or prevents the formation of bland thrombus on TT surfaces. These questions remain clinically important but are not addressed by the available literature. Future prospective studies should explicitly distinguish prophylactic and therapeutic anticoagulation strategies and evaluate their differential impact on thromboembolic risk and bleeding outcomes in patients with TT.

Survival outcomes across all included studies were predominantly determined by tumor-related factors rather than by anticoagulation exposure. Neither cohort studies nor case reports demonstrated a survival advantage associated with anticoagulant therapy (14–21). In RCC, anticoagulated patients exhibited higher mortality; however, this association was confounded by more advanced disease and greater thrombus burden (15). In HCC and other malignancies, survival correlated with tumor stage, extent of vascular invasion, and feasibility of definitive tumor-directed treatment rather than with anticoagulant use (8, 14). Based on available evidence, anticoagulation does not appear to alter the natural history of TT or the oncologic trajectory of affected patients.

In contrast, bleeding risk emerged as a consistent and clinically important safety signal. Anticoagulation was associated with a significantly increased risk of major bleeding in RCC cohorts (15), and clinically relevant bleeding events were reported across tumor types (21). Although HCC cohorts appeared to demonstrate lower rates of variceal bleeding among anticoagulated patients, this finding was likely attributable to aggressive prophylactic variceal management rather than a protective effect of anticoagulation itself (14). The absence of major bleeding in case reports must be interpreted cautiously, given limited follow-up and strong selection bias. Overall, the balance of evidence suggests that anticoagulation exposes patients with TT to substantial harm without demonstrable disease-modifying benefit.

Importantly, the behavior of TT is increasingly influenced by modern systemic anticancer therapies, which may induce tumor regression—including intravascular components—independent of anticoagulation. Targeted agents such as tyrosine kinase inhibitors (e.g., sunitinib, cabozantinib) and immune checkpoint inhibitors have demonstrated activity against RCC and HCC and may contribute to regression of vascular tumor invasion in selected patients (22–25). In this context, apparent thrombus regression reported in isolated cases is difficult to attribute to anticoagulation when administered concurrently with effective systemic therapy. Conversely, anti-angiogenic agents, including bevacizumab and VEGF pathway inhibitors, are associated with increased bleeding risk and may further shift the anticoagulation risk–benefit balance in patients with TT (26–29). These considerations underscore the need for multidisciplinary evaluation and integration of oncologic treatment plans when anticoagulation is contemplated.

These findings reinforce the concept that TT represents a spectrum of malignant intravascular invasion with shared biological features but distinct anatomical and clinical manifestations. Although contiguous venous extension in RCC and HCC, intracardiac tumor extension, and pulmonary tumor embolism differ in presentation and prognosis, all are characterized by malignant tissue occupying the vascular lumen and demonstrating limited responsiveness to anticoagulation (8, 10, 14–20). Across tumor types and locations, thrombus regression attributable solely to anticoagulant therapy was not observed, and apparent thrombus resolution was consistently associated with tumor-directed or mechanical interventions (16–19). In this context, thrombus regression described in isolated reports is difficult to interpret when anticoagulation is administered concurrently with definitive tumor-directed therapy.

Importantly, the current literature does not exclude the possibility that anticoagulation may provide benefit in narrowly defined clinical contexts, such as in patients with mixed malignant and bland thrombotic components or those with concurrent VTE. However, these subgroups were not systematically evaluated, and available data are insufficient to draw definitive conclusions (14, 15, 20). The overall certainty of evidence remains low due to retrospective study designs, small sample sizes, heterogeneity in tumor types and thrombus extent, and confounding by tumor stage and treatment (14–20).

These findings highlight the importance of distinguishing isolated TT from clinical scenarios in which anticoagulation is routinely indicated regardless of tumor involvement, including confirmed PE, proximal DVT, atrial fibrillation with elevated thromboembolic risk, or catheter-associated thrombosis (1, 11, 12). In such cases, anticoagulation should be guided by established principles for cancer-associated thrombosis, with careful consideration of bleeding risk.

From a clinical perspective, tumor-directed strategies, including surgical thrombectomy, locoregional therapies, and systemic anticancer treatment, remain the cornerstone of TT management (8, 11). When used, anticoagulation should be considered an adjunctive, indication-driven therapy rather than a disease-modifying therapy. Multidisciplinary collaboration among oncology, surgery, cardiology, hepatology, and thrombosis specialists is essential to individualize care and avoid unnecessary harm.

## Clinical implications

- Routine anticoagulation for isolated TT is not supported by current evidence. Available data do not demonstrate consistent benefit in thrombus regression, embolic risk reduction, or survival, while a clinically meaningful bleeding risk has been observed, particularly in RCC-associated TT (14–20).
- Tumor-directed strategies remain the cornerstone of management. Surgical thrombectomy, locoregional therapies, and systemic anticancer treatment should be prioritized whenever feasible, as these interventions help control thrombus and improve clinical outcomes (8, 10).
- Anticoagulation should be reserved for conventional indications. These include confirmed bland venous thromboembolism, atrial fibrillation with elevated thromboembolic risk, mechanical heart valves, or catheter-associated thrombosis, and should follow established cancer-associated thrombosis guidelines (1, 11, 12).
- Multidisciplinary evaluation is essential. Management should be individualized through collaboration among oncology, surgery, cardiology, hepatology, and thrombosis specialists, particularly in patients with advanced disease, portal hypertension, or high bleeding risk.
- Empirical anticoagulation for incidentally detected or asymptomatic tumor thrombus should be avoided. In palliative or frail patients, bleeding complications may outweigh any uncertain benefit.

## Conclusion

In summary, available evidence suggests that anticoagulation does not appear to meaningfully modify tumor thrombus biology, induce radiographic regression, reduce thromboembolic recurrence, or improve survival in patients with solid tumor–associated tumor thrombus, while exposing patients to a clinically relevant risk of bleeding. Tumor-directed and mechanical interventions remain the primary determinants of clinical outcomes. Anticoagulation should therefore not be used routinely for isolated tumor thrombus, but may be appropriate when conventional indications are present, such as confirmed pulmonary embolism, proximal deep-vein thrombosis, atrial fibrillation with elevated thromboembolic risk, or catheter-associated thrombosis. Given the low certainty and heterogeneity of existing data, prospective multicenter studies are needed to better define patient subgroups that may derive benefit and to inform evidence-based clinical guidance.

## Data Availability

The original contributions presented in the study are included in the article/**Supplementary Material**. Further inquiries can be directed to the corresponding author.
