# Supplementary material for: Anticoagulant outcomes in managing tumor thrombus: a systematic review
Source: Front Oncol. 2026 Feb 23;16:1773327. doi: 10.3389/fonc.2026.1773327 (PMC12967944; doi:10.3389/fonc.2026.1773327)
Supplement: Supplementary file 1 [file Supplementaryfile1.docx]

**Supplementary Material**

**Search Strategies**

**PubMed / MEDLINE**

- Concept 1: Tumor thrombus

("tumor thrombus"[tiab] OR "tumour thrombus"[tiab] OR "tumor thrombosis"[tiab] OR

"tumour thrombosis"[tiab] OR "cancer thrombus"[tiab] OR "malignant thrombus"[tiab] OR

(("tumor"[tiab] OR "tumour"[tiab] OR cancer*[tiab] OR neoplasm*[tiab])

AND (thrombus[tiab] OR thrombosis[tiab])

AND (invasion[tiab] OR extension[tiab] OR intravascular[tiab] OR venous[tiab])))

OR (("Neoplasms"[Mesh]) AND "Thrombosis"[Mesh])

- Concept 2: Anticoagulants

("Anticoagulants"[Mesh] OR "Anticoagulants"[Pharmacological Action] OR

anticoagulan*[tiab] OR heparin[tiab] OR "Low Molecular Weight Heparin"[Mesh] OR

dalteparin[tiab] OR enoxaparin[tiab] OR fondaparinux[tiab] OR warfarin[tiab] OR

coumarin*[tiab] OR "vitamin K antagonist*"[tiab] OR

"direct oral anticoagulant*"[tiab] OR DOAC*[tiab] OR NOAC*[tiab] OR

apixaban[tiab] OR rivaroxaban[tiab] OR edoxaban[tiab] OR dabigatran[tiab])

- Combine

#1 AND #2

- Optional filters

AND (Humans[Mesh]) AND (Adult[Mesh])

((("tumor thrombosis"[tiab] OR "tumour thrombosis"[tiab] OR "tumor thrombus"[tiab] OR "tumour thrombus"[tiab] OR "intravascular tumor"[tiab] OR "intravascular tumour"[tiab] OR "malignant venous invasion"[tiab] OR "venous tumor invasion"[tiab] OR "IVC thrombus"[tiab] OR "IVC invasion"[tiab] OR "renal vein thrombus"[tiab] OR "renal vein invasion"[tiab] OR "portal vein tumor thrombus"[tiab] OR "hepatic vein tumor thrombus"[tiab] OR ((RCC OR "renal cell carcinoma"[tiab] OR HCC OR "hepatocellular carcinoma"[tiab]) AND (IVC[tiab] OR "inferior vena cava"[tiab] OR "renal vein"[tiab] OR "portal vein"[tiab] OR "hepatic vein"[tiab]) AND (invasion[tiab] OR thrombus[tiab] OR thrombosis[tiab])))) NOT (("bland thrombus"[tiab] OR "bland thrombosis"[tiab]))) AND (("Anticoagulants"[Mesh] OR anticoagulant*[tiab] OR heparin*[tiab] OR LMWH[tiab] OR "low molecular weight heparin"[tiab] OR enoxaparin[tiab] OR dalteparin[tiab] OR fondaparinux[tiab] OR warfarin[tiab] OR coumadin[tiab] OR "vitamin K antagonist*"[tiab] OR VKA[tiab] OR DOAC*[tiab] OR NOAC*[tiab] OR "direct oral anticoagulant*"[tiab] OR apixaban[tiab] OR rivaroxaban[tiab] OR edoxaban[tiab] OR dabigatran[tiab]))

**Cochrane Library**

#1 (tumor* OR tumour* OR cancer* OR malignan* OR neoplasm*) near/3 (thrombus OR thrombosis OR clot*):ti,ab,kw

#2 (anticoagula* OR heparin OR dalteparin OR enoxaparin OR fondaparinux OR warfarin OR

apixaban OR rivaroxaban OR edoxaban OR dabigatran OR "direct oral anticoagulant*" OR

doac* OR noac*):ti,ab,kw

#3 #1 AND #2

#4 Apply filter: Adults (MeSH descriptor: [Adult] explode all trees)

**Embase (via Ovid)**

1. neoplasm thrombosis/ OR ((tumor* OR tumour* OR cancer* OR malignan* OR neoplasm*) adj3

(thrombus OR thrombosis OR clot*)).ti,ab,kw.

2. anticoagulant agent/ OR anticoagulant therapy/ OR

(anticoagula* OR heparin OR dalteparin OR enoxaparin OR fondaparinux OR warfarin OR

coumarin* OR apixaban OR rivaroxaban OR edoxaban OR dabigatran OR

"direct oral anticoagulant*" OR doac* OR noac*).ti,ab,kw.

3. 1 AND 2

4. limit 3 to human

5. limit 4 to adult (18 yrs +)
